# Preparation of high‐quality concentrated fragrance flaxseed oil by steam explosion pretreatment technology

**DOI:** 10.1002/fsn3.1505

**Published:** 2020-03-18

**Authors:** Gaiwen Yu, Tingting Guo, Qingde Huang, Xunwang Shi, Xin Zhou

**Affiliations:** ^1^ Oil Crops Research Institute Chinese Academy of Agricultural Sciences Wuhan China; ^2^ Hubei Key Laboratory of Lipid Chemistry and Nutrition Wuhan China; ^3^ Oil Crops and Lipids Process Technology National & Local Joint Engineering Laboratory Wuhan China

**Keywords:** concentrated fragrance, flaxseed oil, micronutrient content, pretreatment technology, steam explosion

## Abstract

In this study, flaxseed was pretreated by steam explosion technology and subsequently pressed to prepare flaxseed oil. GC, UPLC, HPLC, and GC‐MS techniques were used to analyze the quality characteristics of the prepared flaxseed oil. These included the food safety risk indices, micronutrient components, and oxidative stability. The effects of different steam explosion pressures on the quality characteristics and relative volatile components of flaxseed oil were also investigated. The results revealed that steam explosion pretreatment technology could significantly increase the oil yield, improve micronutrient content, and strengthen the oxidation stability of the product. Moreover, the food safety risk indices (e.g., benzopyrene) were controlled within a reasonable range, while the fatty acid content remained almost unchanged. Notably, the relative pyrazine content in the total volatile components of flaxseed oil was 68.25% when the steam explosion pressure reached 1.2 MPa. This was considered as the main factor that contributed to the unique concentrated fragrance of the produced flaxseed oil. To prove the superiority of the steam explosion pretreatment, we compared this technique with traditional high‐temperature roasting and popular microwave pretreatment techniques. The results revealed that flaxseed oil prepared by steam explosion pretreatment displayed the best quality characteristics and most concentrated fragrance. Thus, steam explosion technology shows great potential for application to produce high‐quality concentrated fragrance flaxseed oil. This study provides significant reference and guidance for the preparation process of flaxseed oil.

## Graphical abstract

The objective of the present study was to carry out different steam explosion experiments on flaxseed, compared the lipid characteristics (physicochemical parameters, fatty acid composition, phytochemicals (tocopherols, phytosterols, polyphenols, flavonoids), oxidation induction time (OIT), and relative volatile components. The results of this study will not only help factories produce high‐quality and concentrated fragrance pressed flaxseed oil, but also have important reference significance for the development of oil crop preparation process.

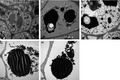

## 1 INTRODUCTION

Flaxseed oil, which is rich in α‐linolenic acid, provides the correct balance of essential fatty acids in the human diet and is gaining increasing popularity as a cooking vegetable oil (Chirino‐Galindo, Barrera‐Argüelles, Trejo‐González, Mejía‐Zepeda, & Palomar‐Morales, 2017; Jiajie & Ma, 2014; Morshedzadeh et al., 2019). Moreover, numerous studies have revealed that a dietary intake of flaxseed oil rich in α‐linolenic acid helps prevent chronic subhealth symptoms such as hyperlipidemia and hyperglycemia (El‐Waseif, El‐Dayem, Hashem, & El‐Behairy, 2014; Kaplan, Şingirik, Erdoğan, & Doran, 2017; Wong, Chahal, Manlhiot, Niedra, & Mccrindle, 2013). Currently, traditional high‐temperature roasting pretreatment is the most commonly used flaxseed oil production process; however, the application of microwave technology to oil crops has also gained popularity in recent years (Koubaa et al., 2016; Nemes, 2012). Yu, Shi, Hong, Huang, and Zhou (2019) pretreated flaxseed at different roasting temperatures with subsequent pressing and analyzed the quality characteristics of the produced flaxseed oils. The results indicated that proper roasting temperature (180–195°C) could provide oils with better flavor and increase oxidation stability. On the other hand, the color of the flaxseed oils became darker and their nutrient components were significantly reduced by improper processing. Yang, Huang, Zhou, Huang, and Deng (2013) extracted and analyzed volatile compounds in both cold‐ and hot‐pressed flaxseed oil samples by using headspace solid‐phase micro‐extraction (HS‐SPME) and gas chromatography–mass spectrometry(GC‐MS). The results revealed a rapid drop in the relative alcohol contents of the volatile compounds in the hot‐pressed flaxseed oil, and the characteristic volatile compounds mainly comprised pyrazines, pyrroles, and pyridines, which endowed hot‐pressed flaxseed oil with a unique toasting flavor. Unfortunately, the aromatic heterocyclic compounds and their complexes display toxic effects on human physiological functions; thus, cold‐pressed flaxseed oil had the characteristics of higher food safety. Ren, Zhang, Sun, Duan, and Zhang (2015) studied the effect of microwave pretreatment on the extraction of flaxseed oil and determined the quality characteristics of the flaxseed oils extracted using different radiation intensities and extraction times. The results indicated that the maximum oil yield was obtained when using microwave radiation at 18 W/g for 210 s. Moreover, the transmission electron microscopy (TEM) results revealed that the microstructures of the pretreated samples were modified after microwave pretreatment, while the fatty acid compositions were not affected by different microwave radiation conditions.

Under the influence of traditional eating habits and cultures, edible oils with highly concentrated fragrances are more favored by consumers (Alberdicedeño, Ibargoitia, Cristillo, Sopelana, & Guillén, 2017). Notably, the pretreatment processes currently employed in the production of concentrated fragrance pressed flaxseed oil lead to a number of disadvantages including the loss of micronutrient components, food safety risk indices that exceed the limitation standards, and poor fragrances. However, to date, studies of steam explosion technology to pretreat fresh flaxseed and its effect on the nutritional and volatile components of flaxseed oil are scarce. Steam explosion technology is a physicochemical pretreatment method that transforms thermal energy into mechanical energy and achieves the separation and structural changes of macromolecule substances in components through instantaneous pressure release and expansion under a high temperature and pressure environment (Li et al., 2019; Shi, Li, Li, Cheng, & Zhu, 2019; Zhang et al., 2019). Therefore, the objective of this study was to carry out steam explosion experiments under different pressures (0.4–0.12 MPa) on flaxseed and compare the lipid characteristics (physicochemical parameters and fatty acid composition), phytochemical (tocopherols, phytosterols, polyphenols, and flavonoid) contents, oxidation induction time (OIT), and relative volatile components of the afforded products. The results of this study provide useful guidelines for the industrial production of high‐quality concentrated fragrance pressed flaxseed oil and are also of important reference significance for the development of oil crop preparation processes.

## 2 MATERIALS AND METHODS

### 2.1 Experimental materials and pretreatment

The yellow flaxseed samples (GS‐Y2012) were collected from Yongchang, Gansu Province.

Steam explosion pretreatment was carried out on an XSS‐QPD multifunctional air expander (Wuhan KINHE Food Machinery Co., Ltd.). A certain amount of flaxseed was weighed, the moisture of flaxseed was adjusted to about 10%, when the water quality was balanced, flaxseed was put into the air puffing bin, and the heating temperature by constant temperature system was controlled (about 220°C). When the pressure of the silo reached the set pressure (0.4 MPa, 0.6 MPa, 0.8 MPa, 1.0 MPa, 1.2 MPa), it was within a very short time (0.0875 s) to complete the pressure release to realize steam explosion (Li et al., 2019).

Referring to the documentation (Cao, Huang, Tian, & Deng, 2016), a sealed microwave digestion instrument (maximum power, 4,800 W and frequency, 2,450 MHz; CEM Corporation) was employed to simulate the most common microwave pretreatment process currently in use. Some fresh flaxseed were accurately weighed, pretreated with microwaves technology under 420 W for 4 min at a frequency of 2,450 MHz and then cooled to room temperature. In addition, the fresh flaxseed moisture was adjusted to 12%, when the water quality was balanced; the flaxseed was pretreated under 700 W for 6 min at a frequency of 2,450 MHz and then cooled to room temperature.

Traditional high‐temperature roasting pretreatment was performed on a GSCH‐series multifunction heat roasting machine (Henan Ruiguang Machinery Co., Ltd.); some flaxseed were weighed; then, the temperature of the multifunction heat roasting machine was set through the temperature control function; a short period of time (about 1 min) was stabilized after the roasting machine was heated to 170°C; finally, the weighing flaxseed was roasted for 45 min; and then it, was cooled naturally to room temperature.

### 2.2 Preparation of flaxseed oil

All the flaxseed samples were pressed with a CA59G oil press (IBG GmbH) to produce the oil products. In view of the loss of water in flaxseed under different pretreatment conditions, the moisture content of flaxseed was controlled by adding distilled water to between 6% and 7% and then squeezed; at the same time, the pressing temperature was controlled within 65°C; and the oil phase after filtering was pressed flaxseed oil.

### 2.3 Determinations

#### 2.3.1 Oil yield contents

The oil yield contents were determined according to ISO659.2009, using analytical grade petroleum for gravimetric analysis in Soxhlet apparatus (B‐811, Buchi Labortechnik AG) for 8 hr.

The oil yield content was calculated as:

$$Y=\frac{M_{1}}{M_{2}\times X}\times 100\%$$

where *Y* is the oil yield content (%), *M*_1_ and *M*_2_ are the rapeseed oil and rapeseed masses, respectively (g), and *X* is the rapeseed oil content (%).

#### 2.3.2 TEM analysis

For TEM analysis the flaxseed samples were fixed in 2.5% glutaraldehyde solution at 4°C, washed with phosphoric acid buffer, treated with a Spurr's low‐viscosity epoxy resin embedding medium, and subsequently heated overnight at 70°C. The embedding samples were sliced by an ultra‐thin slicing machine to obtain 70–90 nm slices, dyed with a lead acetate/uranyl acetate solution for 5–10 min, and finally observed under a transmission electron microscopy (QUANTUM, Quantum Science Instruments Trading Co., Ltd).

#### 2.3.3 Physicochemical properties

Physicochemical properties (AV, POV, P‐AV, K_232_, and K_270_) of the test flaxseed oil samples were measured referring to the AOCS official methods (Firestone, 2003). The benzopyrene (BaP) contents of the pressed flaxseed oil samples were determined using a REAGEN™ benzopyrene enzyme‐linked immune reaction test box (Shenzhen Gongjin Technology Co., Ltd).

#### 2.3.4 Tocopherols

The content of tocopherols in flaxseed oil was quantified by using AOCS Official Method Ce 8–89 with slight modifications. A 2 g (accuracy 0.0001 g) of tested oil sample was weighed in a volume of 25 ml and then dissolving in the layer of the hexane through a 0.22‐μm polytetrafluoroethylene filter. The treated samples (20 μl) were measured by high‐performance liquid chromatography (HPLC) (LC‐20A, Shimadzu Corp.) on a SIL100A column (250 × 4.6 mm, 5 μm; GL Sciences Inc.). The flow rate of the mobile phase which was constituted by the mixture of hexane and isopropanol (99.5:0.5, v/v) was 1 ml/min. α‐ and γ‐tocopherols were determined at 292 nm and 298 nm, respectively.

#### 2.3.5 Phytosterols

The phytosterol contents were determined as follows: 10 ml of 2 mol/L KOH in ethanol was used to saponify 0.2 g (accuracy 0.0001 g) of the tested oil samples and 0.5 ml of 0.5 mg/ml 5*α*‐cholestane (internal standard) at 60°C for 60 min; the unsaponifiable compositions was obtained with hexane. The hexane layer was dried over anhydrous sodium sulfate and silylated using 100 µl N,O‐bis (trimethylsilyl) trifluoroacetamide + 1% trimethylchlorosilane (BSTFA + TMCS) at 105°C for 15 min. The mixture was then dissolved in 1 ml hexane for further analysis on an Agilent 6890A gas chromatography system (Agilent) equipped with a DB‐5HT column (30 m × 0.32 mm, 0.1 μm; Agilent). The nitrogen (carrier gas) flow rate was 2.0 ml/min, while the detector and injection temperatures were maintained at 320°C. The oven temperature was programmed as follows: an original temperature of 60°C for 1 min, increased to 310°C at 4°C/min, and maintained at this temperature for 10 min. The split ratio was 25:1, and the injection volume was 1 μl.

#### 2.3.6 Total phenolics

Briefly, about 1.25 g of flaxseed oil was placed in a 10‐ml centrifuge tube, and 1.5 ml of 80% methanol aqueous solution was added and then shaken with HA9‐HMV multivortex mixer (Wuxi Voshin Instruments Manufacturing Co., Ltd) at 4,863 *g* 15 min in the dark. The supernatant was carefully transferred into another tube and the extracted separately with 1.5 ml of 80% methanol aqueous solution, and this process was repeated 3 times.

0.5 ml of the total phenolic extracts was transferred into a 10‐ml colorimeter tube. Subsequently, 0.5 ml of the Folin–Ciocalteu reagent and 5 ml of distilled water were added and then shaken with HA9‐HMV multivortex mixer (Wuxi Voshin Instruments Manufacturing Co., Ltd) at 4,863 *g* 3 min in the dark. Next, 1 ml of saturated sodium carbonate solution was added and brought up to 10 ml by adding distilled water. After 1 hr at room temperature, the absorbance was analyzed at 765 nm by using a TU‐1901 Dual Beam UV–vis spectrophotometer (Beijing Purkinje General Instrument Co., Ltd.). Gallic acid was served as the calibration curve (*Y* = 0.0034*X* + 0.0774, *R*_2_ = 0.9996). All values were stated as mg sinapic acid equivalents (SAE)/100 g flaxseed oil.

#### 2.3.7 Flavones

The extraction method was consistent with that of total phenolics. Next, 1.0 ml of the extracts was transferred into a 25 ml of volumetric flask. Subsequently, 12.5 ml of 60% ethanol aqueous solution and 0.7 ml of 5% NaNO_2_ solutions were added and then shaken with HA9‐HMV multivortex mixer (Wuxi Voshin Instruments Manufacturing Co., Ltd) at 4,863 *g* 6 min in the dark. Subsequently, 0.7 ml of 10% Al(NO_3_)_3_ solution was added, and 6 min later, 5.0 ml of 1 mol/L NaOH solutions and 60% ethanol aqueous solution were added to scale line of volumetric flask. After 15 min at room temperature, the absorbance was analyzed at 510 nm by using a TU‐1901 Dual Beam UV–vis spectrophotometer (Beijing Purkinje General Instrument Co., Ltd.).

#### 2.3.8 Fatty acid

Flaxseed oil samples used sodium methoxide to methylate and were measured by Agilent 7890A gas chromatography system (Agilent) installed with HP‐INNOWAX capillary column (30 m × 0.32 mm, 0.25 μm, Agilent Corp.). All the temperature of the injector and the detector was 260°C. The flow rate of the N_2_ served as carrier gas with a split ratio of 80:1 was set to 1.5 ml/min. The temperature of the oven was programmed as follows: an original temperature of 210°C for 9 min and then increased to 230°C at the rate of 20°C/min and maintained for 10 min. The injection volume in this experiment was 1 μl. Analysis was performed based on the fatty acid composition in the treated oil samples by comparing the retention time which was determined by the standard fatty acids.

#### 2.3.9 Oxidation induction time

According to the AOCS Cd 12b‐92 method, the OITs of the flaxseed oil samples were determined on a Metrohm Rancimat 743. A 3 g sample was weighed and placed into a glass reaction tube, heated at 110°C, and then passed through 20 L/hr of dried and cleaned air. The volatile organic acid was collected in a measuring vessel comprising 50 ml distilled water. As the oxidation reaction progressed, the water conductivity was automatically measured and the results were reported in hours (hr).

#### 2.3.10 Relative volatile components

According to a previously reported method (Yang et al., 2013), 10 g of the oil sample was weighed, and volatile components were adequately extracted with HS‐SPME instrument (2 cm, 50/30 μm DVB/Carboxen/PDMS, Agilent Technologies) for 10 hr at room temperature and analyzed by using GC‐MS system (Agilent Technologies) installed with a HP‐5MS capillary column (0. 25 μm × 0.25 mm, 30 μm). The oven temperature was programmed as follows: Initial temperature was 38°C, held at 38°C for 1 min, then at a rate of 8°C/min from 38°C to 175°C, then from 175°C to 220°C at a rate of 50°C/min, held at 220°C for 2 min. Split injection was conducted at a split ratio of 10:1, and helium (purity identification, 99.999%) was used as carrier gas at a flow rate of 1.0 ml/min. The spectrometers were measured in electron‐impact (EI) mode, the ionization energy was 70 eV, and the injection temperature and ionization source temperature were 250°C and 230°C, respectively.

#### 2.3.11 Data analysis

All the analyses were implemented in triplicate and presented as the means ± standard errors. Statistical analyses were performed using the SPSS program (SPSS 23.0 for Windows, SPSS Inc.). Duncan's test at the 5% significance level (*p* < .05) and one‐way analysis of variance (ANOVA) were used to determine the significance differences.

## 3 RESULTS AND DISCUSSION

### 3.1 Effect of different pretreatment conditions on the quality characteristics of the pressed flaxseed oil samples

#### 3.1.1 Effect on the yield of pressed flaxseed oil under different pretreatment conditions

**FIGURE 1:**
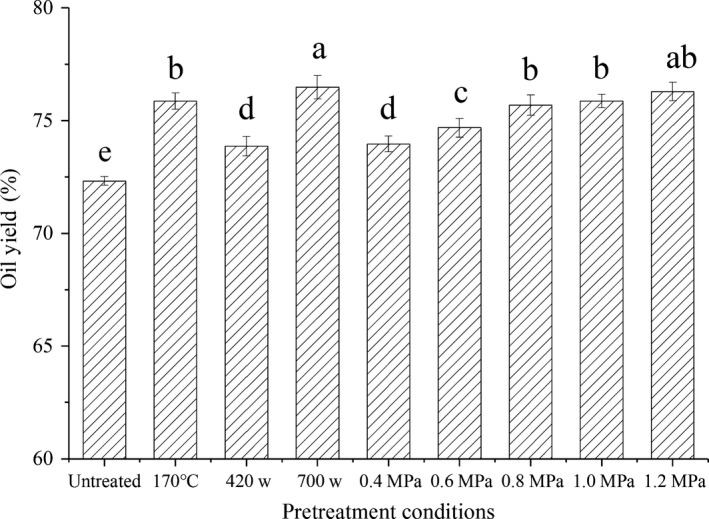
Effect on the yield of pressed flaxseed oil under different pretreatment conditions. Different letters above each column indicate the significant differences (*p* < .05). Conditions: untreated, cold‐pressed flaxseed oil; 170°C, roasted by high‐temperature pretreatment (170°C for 45 min); 420 W, microwave pretreatment (fresh flaxseed; 420 W for 4 min at 2,450 MHz); 700 W, microwave pretreatment (flaxseed moisture adjusted to 17%; 700 W for 6 min at 2,450 MHz); and 0.4–1.2 MPa, pretreated by steam explosion under the indicated pressures

Effect on the yield of pressed flaxseed oil under different pretreatment conditions was illustrated in Figure 1. The results reveal that the oil yield increased with the extension of the explosion pressure range (*p < *.05). Thus, the highest steam explosion pressure (1.2 MPa) afforded the highest flaxseed oil yield (77.5%).

**FIGURE 2:**
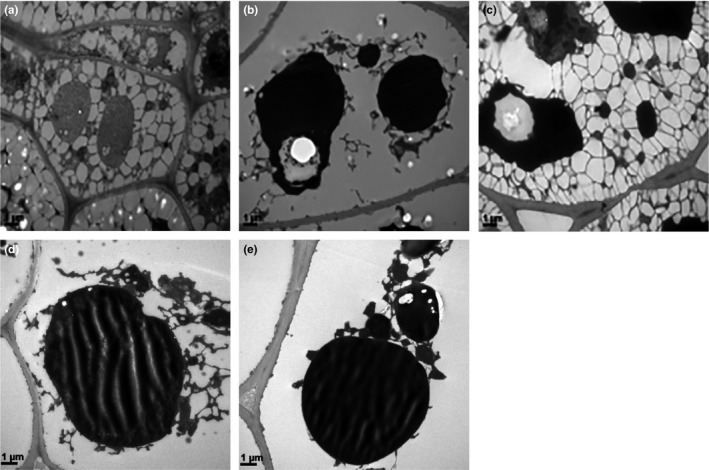
Transmission electron micrographs of the flaxseed samples prepared from different pretreatment conditions (1 μm). (a), untreated cold‐pressed flaxseed oil; (b), flaxseed pretreated by high‐temperature roasting (170°C for 45 min); (c), 420 W, with microwave pretreatment (fresh flaxseed; 420 W for 4 min at 2,450 MHz); (d), 700 W, microwave pretreatment (flaxseed moisture adjusted to 17%; 700 W for 6 min at 2,450 MHz); and (e), steam explosion (1.2 MPa)

Figure 2 presented TEM pictures of the flaxseeds under different pretreatment conditions. We could see clearly the outline of a complete flaxseed cell from pictures, including the cell sap, cytoplasm, and the surface wax layer. It could be concluded that the order of the destructional degree of cell structure was e = d > c > b > a; meanwhile, we found that the destructional degree of flaxseed cell was in good agreement with the oil yield of flaxseed (Figure 1). Analysis suggested that the high destructional degree was attributed to the steam expansion pretreatment technology, the releasing process of this huge energy led to more thorough oil cells structure destruction, the concentrational cell sap and cytoplasm promoted the oil to pack out, and the ruptured surface wax layer reduced oil spill resistance. Thus, a significant increase could be seen in the oil yield (Carneiro, Tonon, Grosso, & Hubinger, 2013).

#### 3.1.2 Main food safety risk indices of the flaxseed oil samples subjected to different pretreatment conditions

Main food safety risk indices of the flaxseed oil samples subjected to different pretreatment conditions are presented in Table 1. The food safety risk indices, including the AVs, POVs, and BaPs, slightly increased following steam explosion treatment. However, these increases were far below the limitation standards. Moreover, the main food safety risk indices were significantly lower for the steam explosion pretreated samples than those observed for the samples subjected to traditional high‐temperature roasting and popular microwave pretreatments. This was attributed to the extremely short completion time of the steam explosion pretreatment process.

**TABLE 1:** Main food safety risk indices of the flaxseed oil samples subjected to different pretreatment conditions

Quality	Untreated	170°C	420 W	700 W	0.4 MPa	0.6 MPa	0.8 MPa	1.0 MPa	1.2 MPa
AV	0.45 ± 0.00^f^	0.86 ± 0.04^a^	0.56 ± 0.01^d^	0.70 ± 0.03^bc^	0.50 ± 0.04^df^	0.52 ± 0.00^df^	0.65 ± 0.01^c^	0.74 ± 0.04^b^	0.82 ± 0.05^a^
POV	0.78 ± 0.10^e^	1.83 ± 0.08^a^	1.26 ± 0.10^c^	1.54 ± 0.06^b^	1.06 ± 0.04^d^	1.24 ± 0.08^cd^	1.46 ± 0.11^b^	1.55 ± 0.06^b^	1.60 ± 0.07^b^
P‐AV	0.11 ± 0.00^e^	1.38 ± 0.06^a^	0.37 ± 0.02^d^	0.56 ± 0.02^c^	0.32 ± 0.01^d^	0.38 ± 0.02^d^	0.52 ± 0.03^c^	0.58 ± 0.03^c^	0.66 ± 0.03^b^
BaP	ND	1.04 ± 0.05^a^	ND	ND	ND	ND	ND	ND	0.68 ± 0.06^b^
K_232_	2.40 ± 0.02^f^	3.36 ± 0.04^a^	2.48 ± 0.06^f^	2.68 ± 0.08^cd^	2.55 ± 0.06^de^	2.62 ± 0.06^cd^	2.68 ± 0.06^cd^	2.72 ± 0.07^c^	2.99 ± 0.06^b^
K_270_	0.28 ± 0.00^f^	1.42 ± 0.03^a^	0.33 ± 0.01^ef^	0.46 ± 0.03^d^	0.32 ± 0.03^ef^	0.38 ± 0.01^e^	0.46 ± 0.03^d^	0.62 ± 0.04^c^	0.75 ± 0.03^b^

Note

Different letters in the columns indicate the significant differences (*p* < .05).

Conditions: untreated, cold‐pressed flaxseed oil; 170°C, roasted by high‐temperature pretreatment (170°C for 45 min); 420 W, microwave pretreatment (fresh flaxseed; 420 W for 4 min at 2,450 MHz); 700 W, microwave pretreatment (flaxseed moisture adjusted to 17%; 700 W for 6 min at 2,450 MHz); and 0.4–1.2 MPa, steam explosion pretreatment at the indicated pressures.

AV, POV, P‐AV, and BaP, respectively, represent the acid value (mg/g oil), peroxide value (meq. O_2_/kg oil), P‐anisidine value, and benzo[a]pyrene (μg/kg oil).

Abbreviation: ND, not discovered.

#### 3.1.3 Micronutrient contents of the flaxseed oil samples subjected to different pretreatment conditions

Micronutrient contents of the flaxseed oil samples subjected to different pretreatment conditions investigated and are listed in Table 2. The results indicated that the contents of micronutrient components in the pressed flaxseed oil were significantly affected by the different pretreatment processes, particularly changes in the explosion pressure. Thus, the tocopherol, phytosterol, polyphenol, and flavonoid contents in the pressed flaxseed oil significantly increased with the extension of the steam explosion pressure range (*p < *.05). These results were attributed to the instantaneous high energy environment caused by the steam expansion process, which accelerated the fat accumulation process and promoted the dissolution of some fat‐soluble micronutrient components (Primo‐Martín & Deventer, 2011). Moreover, when the steam explosion pressure reached 1.2 MPa, the tocopherol, phytosterol, polyphenol, and flavonoid contents were, respectively, 52.28 mg/100 g, 688 mg/100 g, 56.55 mg/100 g, and 342.18 mg/kg, which were markedly superior to the contents afforded by the traditional high‐temperature roasting and popular microwave pretreatments.

**TABLE 2:** Micronutrient contents of the flaxseed oil samples subjected to different pretreatment conditions (mg/100 g oil)

Quality	Untreated	170°C	420 W	700 W	0.4 MPa	0.6 MPa	0.8 MPa	1.0 MPa	1.2 MPa
Tocopherols	30.82 ± 1.06^f^	45.35 ± 1.13^c^	36.81 ± 0.85^e^	54.16 ± 1.27^a^	40.10 ± 1.98^d^	44.92 ± 1.13^c^	48.84 ± 1.25^b^	52.54 ± 1.70^a^	52.28 ± 0.99^a^
Phytosterols	562.12 ± 16.39^d^	620.26 ± 18.20^c^	578.48 ± 15.54^d^	667.02 ± 0.10^ab^	600.87 ± 20.45^cd^	619 ± 11.10^bc^	638.05 ± 30.64^bc^	660.56 ± 25.14^ab^	688.63 ± 28.08^a^
Polyphenols	10.58 ± 0.42^g^	29.10 ± 1.12^ef^	24.95 ± 0.88^f^	55.27 ± 3.73^ab^	30.12 ± 1.70^e^	39.97 ± 2.40^d^	45.78 ± 3.11^c^	51.70 ± 1.98^b^	56.55 ± 1.13^a^
Flavonoids	220.35 ± 7.07^g^	283.21 ± 9.90^de^	245.26 ± 11.31^f^	340.36 ± 7.07^ab^	270.50 ± 14.14^e^	292.28 ± 15.52^de^	296.54 ± 9.90^cd^	318.82 ± 5.66^bc^	342.18 ± 9.19^a^

Note

Different letters in the columns indicate the significant differences (*p* < .05).

Conditions: untreated, cold‐pressed flaxseed oil; 170°C, roasted by high‐temperature pretreatment (170°C for 45 min); 420 W, microwave pretreatment (fresh flaxseed; 420 W for 4 min at 2,450 MHz); 700 W, microwave pretreatment (flaxseed moisture adjusted to 17%; 700 W for 6 min at 2,450 MHz); and 0.4–1.2 MPa, steam explosion pretreatment at the indicated pressures.

#### 3.1.4 Fatty acid compositions of the flaxseed oil samples subjected to different pretreatment conditions

Fatty acid composition is a significant quality characteristic of pressed flaxseed oil. Fatty acid compositions of the flaxseed oil samples subjected to different pretreatment conditions is illustrated in Table 3. In this research, five main different fatty acids were identified and analyzed. The composition of fatty acids in different flaxseed oil samples was semblable, and the carbon chain was 16–18. Linolenic acid (C18:3) content was found to be the highest in oils (57.38%–57.76%), and it prepared by traditional high‐temperature roasting was slightly higher than others. Linolenic acid is a crucial polyunsaturated fatty acid (PUFA) which helps prevent chronic subhealth symptoms such as hyperlipidemia and hyperglycemia (El‐Waseif et al., 2014; Kaplan et al., 2017; Wong et al., 2013). Next in much are such essential 18‐carbon unsaturated fatty acid as oleic acid (C18:1; 19.28%–19.48%) and linoleic acid (C18:2; 12.82%–13.61%), which are essential and significant for the growth and development of human being. This was in agreement with the conclusions reported by Zou et al. (2017), whereby linolenic acid was the dominating fatty acid in pressed flaxseed oil from several cultivars (about 47.9%–55.80% of total fatty acids), followed by oleic acid and linoleic acid at approximate percentages (18.5%–25.3% and 11.0%–14.5%, respectively).

**TABLE 3:** Fatty acid compositions of the flaxseed oil samples subjected to different pretreatment conditions

Fatty acids	Untreated	170°C	400 W	700 W	0.4 MPa	0.6 MPa	0.8 MPa	1.0 MPa	1.2 MPa
C16:0	4.29 ± 0.08^d^	4.50 ± 0.04^a^	4.43 ± 0.02^ab^	4.30 ± 0.03^d^	4.47 ± 0.04^ab^	4.40 ± 0.03^bc^	4.28 ± 0.01^d^	4.30 ± 0.03^d^	4.32 ± 0.04^cd^
C18:0	5.49 ± 0.03^c^	5.41 ± 0.02^ab^	5.26 ± 0.02^a^	5.43 ± 0.02^ab^	5.25 ± 0.08^a^	5.33 ± 0.03^ab^	5.37 ± 0.06^b^	5.45 ± 0.06^bc^	5.46 ± 0.04^bc^
C18:1	19.40 ± 0.05^ab^	19.43 ± 0.06^ab^	19.36 ± 0.06^bc^	19.48 ± 0.02^a^	19.31 ± 0.06^bc^	19.28 ± 0.04^c^	19.36 ± 0.05^bc^	19.35 ± 0.06^bc^	19.36 ± 0.03^bc^
C18:2	13.15 ± 0.10^c^	12.94 ± 0.08^d^	13.51 ± 0.09^ab^	13.18 ± 0.16^c^	13.61 ± 0.20^a^	13.51 ± 0.08^ab^	13.41 ± 0.10^b^	13.44 ± 0.06^ab^	13.48 ± 0.12^ab^
C18:3	57.60 ± 0.09^ab^	57.76 ± 0.06^a^	57.47 ± 0.08^cd^	57.68 ± 0.06^ab^	57.43 ± 0.08^cd^	57.48 ± 0.02^cd^	57.57 ± 0.06^a^	57.38 ± 0.08^d^	57.45 ± 0.07^bc^
SFA	9.78 ± 0.09^ab^	9.90 ± 0.12^a^	9.69 ± 0.08^b^	9.67 ± 0.05^b^	9.73 ± 0.03^b^	9.73 ± 0.06^b^	9.71 ± 0.06^b^	9.75 ± 0.10^b^	9.76 ± 0.08^b^
MUFA	19.40 ± 0.05^ab^	19.43 ± 0.06^ab^	19.36 ± 0.06^bc^	19.48 ± 0.02^a^	19.31 ± 0.06^bc^	19.28 ± 0.04^c^	19.36 ± 0.05^bc^	19.35 ± 0.06^bc^	19.36 ± 0.03^bc^
PUFA	70.75 ± 0.15^cd^	70.70 ± 0.08^d^	70.98 ± 0.03^ab^	70.86 ± 0.06^abc^	71.04 ± 0.06^a^	70.95 ± 0.04^ab^	70.97 ± 0.10^ab^	70.82 ± 0.11^abc^	70.92 ± 0.04^bc^
UFA	90.15 ± 0.20^a^	90.12 ± 0.03^a^	90.34 ± 0.04^a^	90.35 ± 0.06^a^	90.32 ± 0.12^a^	90.23 ± 0.05^a^	90.33 ± 0.15^a^	90.22 ± 0.09^a^	90.28 ± 0.08^a^

Note

Different subscript letters in the columns indicate the significant differences (*p* < .05).

Conditions: untreated, cold‐pressed flaxseed oil; 170°C, roasted by high‐temperature pretreatment (170°C for 45 min); 420 W, microwave pretreatment (fresh flaxseed; 420 W for 4 min at 2,450 MHz); 700 W, microwave pretreatment (flaxseed moisture adjusted to 17%; 700 W for 6 min at 2,450 MHz); and 0.4–1.2 MPa, steam explosion pretreatment at the indicated pressures.

Abbreviations: MUFA, monounsaturated fatty acids; PUFA, polyunsaturated fatty acids; SFA, saturated fatty acids; UFA, unsaturated fatty acids.

Statistical analysis indicated that it had significant differences in fatty acid composition (*p* < .05), while no significant difference was observed in the total content of PUFA (*p* > .05) among the three oil preparation processes. The fatty acid composition of flaxseed oil prepared by steam explosion pretreatment at 1.2 MPa comprised palmitic acid (4.32%), stearic acid (5.46%), oleic acid (19.36%), linoleic acid (13.48%), and linolenic acid (57.45%). Moreover, changes in the steam explosion pressures did not result in any significant changes in the total content of UFA (*p* > .05), and no isomerization or oxidation of fatty acids occurred under different pretreatment conditions.

#### 3.1.5 Effects on the oxidative stability of pressed flaxseed oil prepared under different pretreatment conditions

**FIGURE 3:**
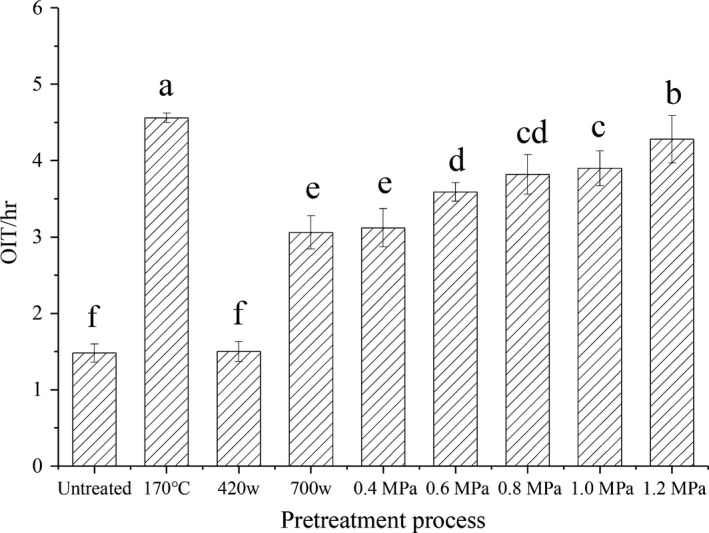
Effects on the oxidative stability of pressed flaxseed oil prepared under different pretreatment conditions. Different letters above each column indicate the significant differences (*p* < .05). Conditions: untreated, cold‐pressed flaxseed oil; 170°C, pretreated by high‐temperature roasting (170°C for 45 min); 420 W, with microwave pretreatment (fresh flaxseed; 420 W for 4 min at 2,450 MHz); 700 W, microwave pretreatment (flaxseed moisture adjusted to 17%; 700 W for 6 min at 2,450 MHz); and 0.4–1.2 MPa, pretreated by steam explosion under the indicated pressures; OIT represents the oxidative induction time

The oxidation stability of edible oil was affected by substances including polyphenols, phytosterols, and tocopherols without exogenous antioxidants (Athira, Nickerson, & Supratim, 2018). The oxidation stabilities of the flaxseed oil samples prepared under different steam explosion pressures are illustrated in Figure 3. Results for the untreated samples and samples subjected to traditional high‐temperature roasting and popular microwave pretreatment were also included. The results indicated that the oxidation stability of flaxseed oil was markedly affected by the different pretreatment processes. Moreover, the oxidation stability of the flaxseed oil was significantly improved with the extension of the steam explosion pressure range (*p* < .05). Thus, when the steam explosion pressure was 1.2 MPa, the OIT of the flaxseed oil was 4.66 hr, which was nearly threefold higher than that of the unpretreated flaxseed oil.

### 3.2 Relative contents of the volatile components of the flaxseed oil samples subjected to different pretreatment conditions

#### 3.2.1 Analysis of the main relative volatile components of flaxseed oil

The relative contents of the volatile components of the flaxseed oil samples subjected to different pretreatment conditions are listed in Table 4. The characteristics of the relative volatile substances were classified into six categories, namely alcohols, aldehydes, acids/ketones, esters, heterocycles, and nitriles. The results clearly revealed differences in the volatile contents of the flaxseed oil prepared from different pretreatment methods. These differences are further discussed below.

**TABLE 4:** Relative contents of the volatile components of the flaxseed oil samples subjected to different pretreatment conditions

Components	Untreated	170°C	420 W	700 W	1.2 MPa
1‐Pentanol	7.80	0.71	8.53	3.10	0.83
1‐Hexanol	21.28	0.85	29.35	13.32	1.54
1‐Octen‐3‐ol	1.19	0.63	8.89	3.47	0.34
1‐Octanol	5.04	ND	ND	0.75	ND
1,3‐Butanediol	ND	ND	7.05	4.56	ND
2‐Nitro‐tertiary butanol	ND	ND	ND	0.12	ND
2‐Heptanol	11.52	ND	5.28	ND	ND
1‐Propanol, 2‐methyl‐	26.04	ND	7.05	5.94	ND
Alcohols	72.87	2.19	66.15	31.26	2.71
2‐Furancarboxaldehyde, 5‐methyl–	ND	3.28	ND	2.30	2.72
Hexanal	3.24	ND	3.68	2.71	ND
Furfural	ND	14.12	ND	ND	4.45
2,4‐Heptadienal, (E,E)‐	ND	2.28	ND	ND	1.22
3,4‐Dimethyl‐benzaldehyde	ND	ND	ND	ND	0.12
2‐Methyl‐butanal	0.58	ND	ND	ND	0.04
Benzeneacetaldehyde	ND	ND	ND	ND	0.39
4‐Methyl‐benzaldehyde	ND	ND	ND	ND	0.50
Aldehydes	3.82	19.68	3.68	5.01	9.44
3‐Hydroxy‐3,5‐dimethyl‐2‐hexanone	0.38	2.06	0.96	1.84	0.66
2,2‐Dimethyl‐cyclobutanone	ND	ND	ND	1.11	0.18
2‐Heptanone	ND	ND	ND	ND	0.39
1‐Hydroxy‐2‐propanone	ND	ND	ND	ND	2.48
2‐methyl‐3‐pentanone	ND	ND	ND	ND	0.46
4‐Hydroxy‐butanoic acid	0.32	1.82	2.42	1.87	0.83
Acetic acid	ND	5.68	ND	ND	0.53
Acids/ketones	0.70	9.61	3.38	4.82	5.53
2(3H)‐Furanone, 5‐Ethyldihydro‐	ND	ND	ND	2.10	0.29
Butyrolactone	3.77	ND	2.93	0.65	1.53
2‐Furanmethanol	ND	2.03	ND	ND	2.53
Esters	3.77	2.03	2.93	2.75	4.35
Pyrazine	ND	1.89	ND	1.08	4.09
Methylpyrazine	ND	15.82	ND	2.62	16.67
2,5‐Dimethyl‐pyrazine	ND	7.37	ND	6.42	9.27
2,6‐Dimethyl‐pyrazine	ND	7.37	ND	5.24	9.29
2,3‐Dimethyl‐pyrazine	ND	1.61	ND	ND	2.03
2‐Ethyl‐6‐methylpyrazine	ND	2.65	ND	0.87	2.37
2‐Ethyl‐5‐methylpyrazine	ND	2.65	ND	1.03	4.34
Trimethylpyrazine	ND	ND	ND	2.19	4.19
3‐Ethyl‐2,5‐dimethyl‐pyrazine	ND	2.01	ND	1.21	6.62
2‐Ethenyl‐5‐methylpyrazine	ND	0.50	ND	ND	0.47
Pyrazinamide	ND	0.71	ND	ND	0.82
1‐(5‐Methyl‐2‐pyrazinyl)‐1‐ethanone	ND	0.22	ND	ND	0.38
Acetylpyrazine	ND	0.61	ND	ND	ND
3‐Ethyl‐thiophene	ND	0.42	ND	ND	1.03
Dihydro‐3‐(2H)‐thiophenone	ND		ND	ND	0.15
2‐Thiophenecarboxaldehyde	ND	0.56	ND	ND	0.72
4,6‐Dimethyl‐pyrimidine	ND	0.11	ND	ND	5.31
1H‐1,2,4‐Triazol‐3‐amine, N‐methyl	ND	3.06	ND	9.55	ND
7‐Methyl‐triazolo(4,3‐b)(1,2,4)‐triazine	ND	0.73	ND	ND	ND
Pyrazines	0	48.29	0	30.21	68.25
Pyrrole	ND	0.99	ND	0.75	4.75
3‐Methyl‐1H‐pyrrole	ND	0.05	ND	1.04	0.15
1‐(1H‐pyrrol‐2‐yl)‐ethanone	ND	0.29	ND	ND	0.17
1H‐Pyrrole‐2‐carboxaldehyde	ND	0.22	ND	ND	0.75
5‐Methyl‐2‐pyridinamine	ND	2.43	ND	ND	ND
5‐Methyl‐2‐furanmethanol	ND	1.40	ND	ND	0.45
2,6‐Dimethyl‐4‐pyridinamine	ND	1.72	ND	ND	ND
2‐Ethyl‐thiophene	ND	1.28	ND	1.04	ND
2‐Ethylfuran	6.32	1.40	4.58	0.75	ND
1‐Methyl‐1H‐pyrrole	ND	0.99	ND	ND	ND
1H‐Pyrrole‐2‐carboxaldehyde,1‐methyl	ND	5.13	ND	ND	ND
2‐Ethyl‐thiophene	ND	1.30	ND	ND	ND
Other heterocyclic compounds	6.32	15.2	4.58	3.58	6.27
2‐Butenenitrile	ND	2.58	ND	2.16	2.52
3‐Butenenitrile	ND	3.26	ND	2.84	3.19
Nitriles	0	5.84	0	5.00	5.71

Note

Conditions: Untreated, cold‐pressed flaxseed oil; 170°C, roasted by high‐temperature pretreatment (170°C for 45 min); 420 W, microwave pretreatment (fresh flaxseed; 420 W for 4 min at 2,450 MHz); 700 W, microwave pretreatment (flaxseed moisture adjusted to 17%; 700 W for 6 min at 2,450 MHz); and 1.2 MPa, steam explosion pretreatment at the indicated pressures.

Abbreviation: ND, not discovered.

#### 3.2.2 Changes in the characteristic volatile substances of flaxseed oil

In a previous study, Yang et al. (2013) have reported that unpretreated flaxseed oil has an inherent fragrance. On the other hand, flaxseed oil prepared by steam explosion pretreatment (1.2 MPa) had a pleasant fragrance. The results in Table 4 revealed that the volatile substances of the unpretreated pressed flaxseed oil mainly comprised alcohols (n‐hexanol, pentanol, and heptanol) that gave the product a fruity aroma. It also contained some esters and aldehydes, such as hexanal, furfural, and butyrolactone. The volatile components of flaxseed oil prepared by steam explosion pretreatment (1.2 MPa) comprised a lower alcohol content, while the pyrazine contents were reported to be as high as 68.25%. This was considered to be the primary cause of the unique concentrated fragrance of flaxseed oil (Müller & Rappert, 2010; Wei, Liu, Xi, Cao, & Huang, 2014).

#### 3.2.3 Changes in the aldehyde and ketone/acid oxidation products

Aldehydes and ketones/acids are products of lipid oxidation. Moreover, flaxseed oil could be easily oxidized at high temperatures because of its high polyunsaturated fatty acid content (Abbasi, Samadi, Jafari, Ramezanpour, & Shams‐Shargh, 2019). The changes in the aldehyde and ketone/acid oxidation products are listed in Table 4. The relative aldehyde and ketone/acid contents in the volatile components of the pressed flaxseed oil prepared by traditional high‐temperature roasting were significantly higher than those of the unpretreated flaxseed oil and presented 4.15‐ and 12.72‐fold increases, respectively. On the other hand, their steam explosion pretreated (1.2 MPa) counterparts only displayed 1.47‐ and 6.90‐fold increases, respectively. Therefore, it could be concluded that steam explosion technology did not lead to the significant fission of pressed flaxseed oil.

#### 3.2.4 Nitrile substances in flaxseed oil

Nitrile substances are rarely found in the volatile substances of vegetable oil. In this study, two types of nitriles, 2‐butenenitrile and 3‐butenenitrile, were detected in the volatile substances of pressed flaxseed oil. According to the literature (Müller & Rappert, 2010), nitrile substances are produced via the decomposition of cyanogenic glucoside, a toxic compound present in flaxseed. Thus, the results suggested that the cyanogenic glucoside present in flaxseed could be decomposed after steam explosion pretreatment, thereby reducing the toxicity of cyanogenic glycosides in the pressed flaxseed meal.

#### 3.2.5 Effect of the volatile substances on the edible safety of flaxseed oil

Studies have indicated that the Maillard reaction could improve the food color and flavor but is accompanied by the production of some harmful substances (Sánchez‐Ortiz, Bejaoui, Quintero‐Flores, Jiménez, & Beltrán, 2018; Zhou et al., 2016). Table 4 revealed that the contents of the harmful volatile substances (e.g., pyrrole, 5‐methyl‐2‐pyridinamine, 2‐ethylfuran) in the pressed flaxseed oil samples prepared by traditional high‐temperature roasting were much higher than those observed in the unpretreated flaxseed oil. On the other hand, it is worth noting that the preparation of flaxseed oil samples by microwave pretreatments produced such relatively few negative substances as aldehyde and ketone/acid oxidation products and other harmful ingredients, while the pyrazine contents were found to be only 0% and 30.21% (400 W, 700 W, respectively), which was inferior to other pretreatment processes. In the steam explosion pretreated samples, the aromatic heterocyclic compounds and their complexes, known for their toxicological effects on human physiological functions, were relatively few. These results suggested that compared to the flaxseed oils prepared via traditional high‐temperature roasting and microwave pretreatments, the steam explosion pretreated flaxseed oils were safer and more concentrated fragrance for human consumption.

## 4 CONCLUSIONS

This study investigated three flaxseed oil pretreatment methods, namely traditional high‐temperature roasting, popular microwave, and steam explosion of flaxseed. The results indicated that the extension of the steam explosion pressure range increased the flaxseed oil yield, improved the micronutrient contents, significantly strengthened the oxidation stability, and controlled the food safety risk indices. When the steam explosion pressure reached 1.2 MPa, the oil yield and tocopherol, phytosterol, polyphenol, and flavonoid contents were 77.5% and 52.28 mg/100 g, 688.63 mg/100 g, 56.55 mg/100 g, and 342.18 mg/kg, respectively. In addition, the food safety risk indices were controlled within a reasonable range, while the effect on the fatty acid contents was insignificant.

Among the volatile components of flaxseed oil prepared by steam explosion pressure (1.2 MPa) pretreatment, the content of the characteristic volatile components (pyrazines) reached 68.25%, which endowed the flaxseed oil with its unique concentrated fragrance. Additionally, the volatile components of these samples comprised relatively few aromatic heterocyclic compounds and their complexes, which are known for their toxic effects on human physiological functions. These results indicated that from the three studied methods, the preparation of pressed flaxseed oil via steam explosion pretreatment presented the best quality characteristics and most concentrated fragrance. Therefore, it would be feasible to utilize steam explosion technology to produce a high‐quality concentrated fragrance pressed flaxseed oil. Moreover, this research provides significant reference and guidance for the preparation process of flaxseed oil.

## CONFLICT OF INTEREST

The authors declared that they had no any conflict of interest.

## ETHICAL APPROVAL

The study did not include any animal or human tests.
